# Oral Immunization Against Candidiasis Using *Lactobacillus casei* Displaying Enolase 1 from *Candida albicans*

**DOI:** 10.3797/scipharm.1404-07

**Published:** 2014-07-23

**Authors:** Seiji Shibasaki, Miki Karasaki, Senji Tafuku, Wataru Aoki, Tomomitsu Sewaki, Mitsuyoshi Ueda

**Affiliations:** ^1^ General Education Center and Graduate School of Pharmacy, Hyogo University of Health Sciences, 1-3-6 Minatojima, Chuo-ku, Kobe 650-8530, Japan.; ^2^ Genolac BL Corporation, Okinawa Industry Support Center 4F, 1831-1, Oroku, Naha City, Okinawa 901-0152, Japan.; ^3^ Division of Applied Life Sciences, Graduate School of Agriculture, Kyoto University, Kitashirakawa-oiwakecho, Sakyo-ku, Kyoto 606-8502, Japan.

**Keywords:** Eno1p, *Candida albicans*, *Lactococcus casei*, Molecular display technology, Candidiasis

## Abstract

Candidiasis is a common fungal infection that is prevalent in immunocompromised individuals. In this study, an oral vaccine against *Candida albicans* was developed by using the molecular display approach. Enolase 1 protein (Eno1p) of *C. albicans* was expressed on the *Lactobacillus casei* cell surface by using poly-gamma-glutamic acid synthetase complex A from *Bacillus subtilis* as an anchoring protein. The Eno1p-displaying *L. casei* cells were used to immunize mice, which were later challenged with a lethal dose of *C. albicans*. The data indicated that the vaccine elicited a strong IgG response and increased the survival rate of the vaccinated mice. Furthermore, *L. casei* acted as a potent adjuvant and induced high antibody titers that were comparable to those induced by strong adjuvants such as the cholera toxin. Overall, the molecular display method can be used to rapidly develop vaccines that can be conveniently administered and require minimal processing.

## Introduction

Candidiasis is a common infection in immunocompromised individuals and is caused by the opportunistic fungal pathogen, *Candida albicans* or by other *Candida* species [1–5]. Candidiasis diagnosis is challenging due to the unavailability of rapid and efficient detection assays. Candidiasis is commonly treated by administering antifungal agents such as caspofungin, micafungin, anidulafungin, and amphotericin B [6]. However, the emergence of drug-resistant *Candida* strains has rendered some treatments ineffective. Therefore, the development of vaccines against candidiasis is of considerable research interest.

Previous studies have identified several candidate *C. albicans* proteins for vaccine development. For example, mice vaccinated with enolase 1 protein (Eno1p), a glycolytic enzyme of *C. albicans* encoded by the *ENO1* gene, showed increased antibody titers against Eno1p and higher survival time than mice that were not vaccinated [7]. In addition to Eno1p, other *C. albicans* proteins such as the hyphal wall protein, glyceraldehyde-3-phosphate dehydrogenase, phosphoglycerate kinase [8], and malate dehydrogenase 9 can also induce protective immune responses against candidiasis when administered with appropriate adjuvant compounds. In vaccine development studies, a convenient way to administer antigens can accelerate research.

In recent years, several molecular display approaches that use genetically engineered microorganisms to produce foreign proteins on the cell surface have been developed [10–12]. The molecular display method involves the fusion of a heterologous protein, such as an antigen, with a bacterial cell-wall protein to enable transport and anchoring of the hybrid protein to the cell surface. The molecular display method has been used to express various functional and antigenic proteins on the microbial cell surface. For example, an antigen obtained from the red sea bream iridovirus (RSIV), a fish pathogen, was displayed on the yeast cell surface to create an orally administered vaccine for cultured fish [13]. Molecular display of HPV16 E7 on *Lactobacillus casei* with the cell-wall anchor of the *Streptococcus pyogenes* M6 protein has been reported to induce cellular immunity against HPV in mice [14]. Another study used the molecular display method to express the HPV16 E7 antigen on the surface of *L. casei* by using poly-gamma-glutamic acid (γ-PGA) synthetase complex A (PgsA) from *Bacillus subtilis* as an anchoring protein [15]. The study showed that mice orally inoculated with this vaccine showed E7-specific antitumor immune responses [15]. Thus, *L. casei* has been previously used in oral vaccine development and is a Generally Recognized As Safe (GRAS) organism [16]. Furthermore, *L. casei* can be used for vaccine preparation without an extensive purification process as required for vaccines that use pathogenic organisms such as *Escherichia coli*. In addition, molecular display methods produce antigenic proteins more rapidly and conveniently than the conventional vaccine production methods.

In this study, we used a molecular display method to express a *C. albicans* antigen, Eno1p, on *L. casei* and developed a novel type of oral vaccine against candidiasis. The Eno1p from *C. albicans* was selected as the model antigen for display on *L. casei* because the vaccine effect of Eno1p has been demonstrated using a molecular display system involving *Saccharomyces cerevisiae* cells [17]. Delivery of the cells to mice by oral administration prolonged the survival of mice infected with lethal levels of *C. albicans*.

These findings indicate that the *L. casei* display system might provide a convenient tool to develop vaccines against candidiasis and other fungal diseases.

## Results and Discussion

### Construction of the Plasmid for Display of Eno1p on L. casei

**Fig. 1.**
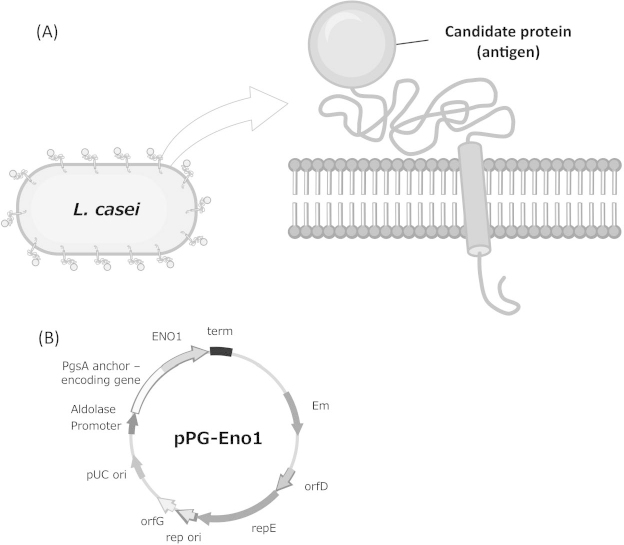
Schematic illustration of the cell surface display of a protein on *Lactobacillus casei* and its genetic construction (A) Molecular display of a candidate protein on the *L. casei* cell surface. (B) The plasmid pPG-Eno1 for the display of a candidate protein (in this case, Eno1p) on the surface of *L. casei* with poly-gamma-glutamic acid (γ-PGA) synthetase complex A (PgsA) as an anchoring protein.

The amplification of the *C. albicans Eno1* gene by PCR and using pULD1-eno1 17 as a template yielded a fragment of the expected size, which was cloned into the pKV plasmid. The insert within pKV was sequenced and compared with the appropriate sequences in the *Candida* genome database (http://www.candidagenome.org/). The plasmid was named pPG-eno1 (Fig. 1B) and introduced into *L. casei* 525 to display the antigenic protein on its surface (Fig. 1A). Poo et al. suggested that PgsA could successfully display the antigenic protein on *L. casei* and the cell induced a mucosal immune response [15]. Therefore, PgsA was selected as the anchoring protein for Eno1p.

### Preparation of Eno1p- Displaying L. casei for Oral Administration

**Fig. 2.**
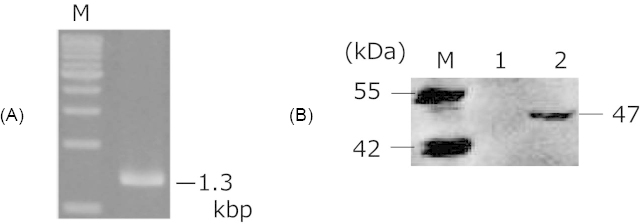
Confirmation of Eno1p display (A) Agarose gel electrophoresis of the PCR-amplified *ENO1* sequence. (B) Western blot analysis of Eno1p. Lane M: molecular weight marker; lane 1: cell surface fraction of control cell (*L. casei* 525); lane 2: cell surface fraction of *L. casei* harboring pPG-Eno1.

Plasmid transfection was confirmed by performing auxotrophic selection and colony direct PCR. The colony PCR yielded a 1.3-kbp fragment of the size of the *ENO1* gene (Fig. 2A). The Eno1 proteins (Eno1p) were identified using western blotting. The anti-FLAG antibody detected a protein band at the molecular weight of 47 kDa, which is the expected Eno 1 protein size (Fig. 2B). Taken together, the genetic and protein analyses data indicated that Eno1p-displaying *L. casei* cells were successfully constructed.

### Determination of C. albicans Lethal Dose

**Fig. 3.**
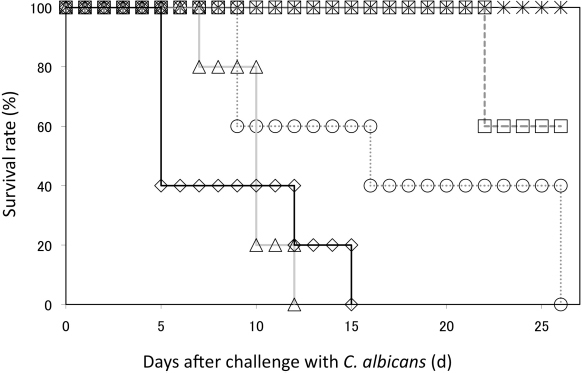
*C. albicans* challenge test *C. albicans* strain SC5314 was injected into mice and the survival rate was evaluated. Asterisk: 2.2 × 10^3^ cells; square: 1.8 × 10^4^ cells; circle: 8.8 × 10^4^ cells; triangle: 2.2 × 10^5^ cells; diamond: 2.2 × 10^6^ cells.

To determine the lethal dose of *C. albicans* for C57BL/6 mice, various concentrations of *C. albicans* SC5314 cells that ranged between 1.0 × 10^3^ to 5.0 × 10^5^ cells were injected into the mice as described previously [18, 19]. A dosage of 2.2 × 10^5^ cells per animal was considered as standard and doses that were lower or higher than the standard dose were assessed to determine the lethal dose (Fig. 3). The data indicated that the LD_50_ was 1.9 × 10^4^ cells/mouse (Fig. 3). On the 12^th^ day after *C. albicans* inoculation, the survival rate of mice that were administered 2.2 × 10^5^ cells became 0%. Therefore, administering a *C. albicans* dose of 1.1 × 10^5^ cells/mouse was thought to be enough to kill all the mice with no immunization.

### Immunological Response after Administration of L. casei Cells

**Fig. 4.**
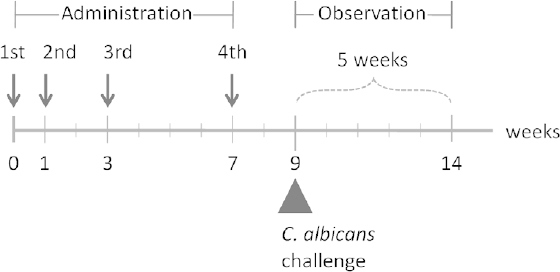
Schedule of oral administration of Eno1p-displaying *L. casei*

**Fig. 5.**
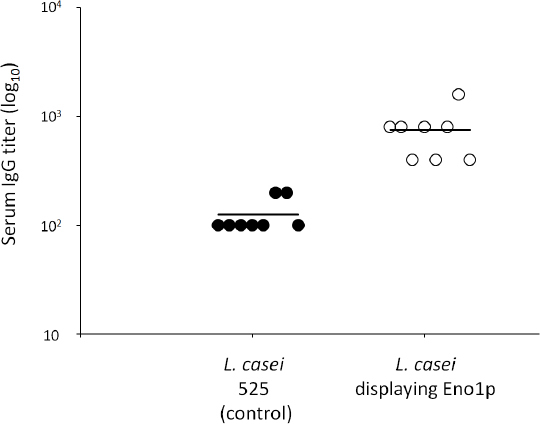
Antibody response of mice after oral administration of *L. casei* cells

The mice were vaccinated by oral administration of the *L. casei* 525 cells (control) or Eno1p-displaying *L. casei* cells four times during the 7-week period (Fig. 4). The average anti-Eno1p antibody titer generated after oral administration of Eno1p-displaying *L. casei* cells was 7.5 × 10^2^ and that of the control *L. casei* 525 was 1.3 × 10^2^ (Fig. 5). In the case of Eno1p-displaying yeast [17], the antibody titer (5.2 × 10^3^) was seven times higher than that of Eno1p-displaying *L. casei*. A comparison of the average antibody titers generated by *L. casei* and yeast indicated that yeast induced a better immunological response against Eno1p [17]. However, there was less variation in antibody titers generated by Eno1p-displaying *L. casei* (titer range: 1.0 × 10^2^ to 1.6 × 10^3^). The antibody titers generated by Eno1p-displaying yeast showed more variability (titer range: 1.0 × 10^2^ to 5.2 × 10^4^). Thus, the antibody immune response generated by *L. casei* may be advantageous for generating vaccines. In addition, *L. casei* cells are potent adjuvants and the control mice inoculated with *L. casei* cells showed a 1.3 × 10^2^ IgG titer, which was comparable to the titer of mice inoculated with adjuvant compounds, Freund’s incomplete adjuvant, and the cholera toxin (approximately 2 × 10^2^ IgG titer) [17]. The adjuvant characteristics of *L. casei* have been previously reported 20 and cell wall components such as beta-glucan have been identified as potent adjuvants in *S. cerevisiae* [21].

### Vaccine Efficacy of Orally Administrated Eno1p-Expressing L. casei Cells

Although the direct quantification of Eno 1p displayed on the *L. casei* surface could not be performed before the oral vaccination of mice, we used a previously published methodology for estimating Eno 1p in a yeast display system [17]. *L. casei* cell growth was measured to indirectly estimate Eno 1p expression. The data indicated that the cell growth pattern of *L. casei* with and without Eno1p display was similar to that of *S. cerevisiae* cultured in liquid medium and that both *L. casei* and *S. cerevisiae* cells progressed into the late log phase after 24 h of cultivation. Since *S. cerevisiae* cultivated for 24 h yielded the largest number of Eno1p molecules on the cell surface, *L. casei* cells grown in liquid medium for 24 h were harvested and used for the vaccination. Mice were orally administered four doses of *L. casei* cells over the course of a 7-week period before challenging with a lethal dose of *C. albicans*.

The survival rate data indicated that after the challenge with *C. albicans* for 25 d, 20% of the mice that received an oral administration of the Eno1p-displaying cells survived longer than the mice that received an oral administration of the control cells (Table 1). After 35 d post-challenge, 10% of the mice that received an oral administration of the Eno1p-displaying cells had survived. Although this survival rate is lower than that of the Eno1p-displaying yeast cells that showed a 60% survival rate in a previous study [17], increasing the administered dose of *L. casei* may enhance the induction of the immune response [22]. These results indicate that a molecular display system using *L. casei* and the antigenic protein Eno1p can be used to develop an effective oral vaccine against candidiasis. To enhance the survival rate by vaccination, the efficiency of the transformation of *L. casei* should be analyzed and improved further.

**Tab. 1.** Survival rate of *C. albicans*-infected mice that were immunized with either control *L. casei* or Eno1p-displayed *L. casei*

Days	*L. casei* 525 control cells (%)	Eno1p-displayed *L. casei* cells (%)
0	100	100
5	100	100
15	50	70
25	0	20
35	0	10

## Conclusion

**Fig. 6.**
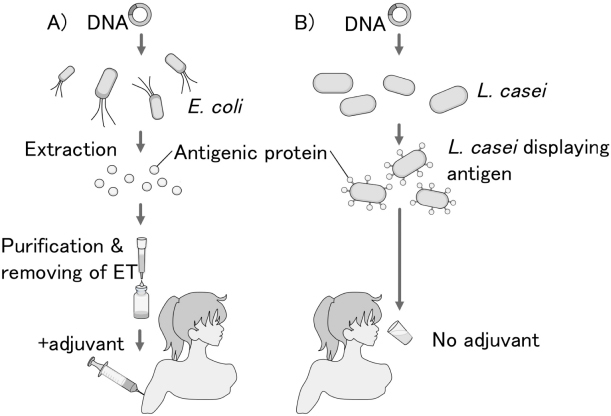
Schematic comparison of development and administration procedures of conventional vaccine and oral vaccine produced by the molecular display of an antigen on the microorganism’s cell surface (A) Conventional vaccination using proteins produced by *E. coli* requires antigen purification and endotoxin (ET) removal. (B) Oral vaccine produced by the molecular display system does not require intensive processing.

In summary, *C. albicans* Eno1p was successfully displayed on the surfaces of *L. casei* cells. Mice immunized with the Eno1p-displaying *L. casei* cells had enhanced survival rates after exposure to a lethal dose of *C. albicans*. The oral administration of the *L. casei* cells displaying Eno1p on their surfaces protected 20% of the mice against candidiasis. The present study demonstrates that the molecular display method can be used for expressing immunogenic proteins on bacterial cells for developing oral vaccines against candidiasis and other infectious diseases. Molecular display system–based vaccine development is rapid and simply requires the DNA sequence encoding the antigenic protein and the appropriate host cells. This vaccine does not require protein purification, which is a tedious process required for conventional vaccine development (Fig. 6). The molecular display methodology can therefore be efficiently adapted to rapidly develop vaccines against other infectious diseases and emerging pandemics. For the development of additional vaccines against candidiasis, we have used proteomic analyses to identify other candidate antigenic proteins [23, 24] and characterized various host cells that could be suitable for developing a molecular display system [25].

## Experimental

### Materials and Methods

#### Strains and Media

The *E. coli* strain DH5α [F^−^ *endA1 hsdR17* (r_K_^−^, m_K_^+^) *supE44 thi1 recA1 gyrA96 ΔlacU169 ðφ80 lacZΔM15*] was used as a host for the manipulation of recombinant DNA. The *E. coli* strain BL21 [F^−^ *ompT hsdSB*(r_B_^−^, m_B_^−^) *gal dcm* (DE3)] was used to produce the antigenic protein. Both *E. coli* strains were grown in LB medium containing 1% (w/v) tryptone, 0.5% (w/v) yeast extract, 0.5% (w/v) sodium chloride, and 0.1% (w/v) glucose. *L. casei* strain 525 was cultured in MRS medium (Becton Dickinson, Franklin Lakes, NJ, USA).

#### Plasmid Construction and Transformation of Microbial Cells

The plasmid pPG-eno1 for the display of Eno1p on the *L. casei* cell surface was constructed as described in this section. First, the *C. albicans* Eno1p-encoding plasmid pULD1-eno1 17 was propagated in *E. coli.* Next, the *ENO1* gene was amplified by performing PCR with the following two primers:

5′-CGGGATCCATGTCTTACGCCACTAAAATCCAC-3′ and 5′-GCTCTAGATTACAATTGAGAAGCCTTTTGGAAATCTTTACC-3′

The *ENO1* PCR product was inserted into pKV by using the BamH1 and Xba1 restriction sites [22]. The nucleotide sequence of this construct was assessed by using an ABI PRISM 3130 Genetic Analyzer (Applied Biosystems, Foster City, CA, USA). The constructed plasmid pPG-Eno1 was transformed into *L. casei 525* by using the previously described electroporation protocol for microorganisms [26].

#### Colony PCR and Western Blot Analysis

To assess the transformation of *L. casei* with the recombinant plasmid, select colonies were used to perform PCR with primers flanking a region on the PgsA-Eno1-FLAG sequence. For western blot analysis, the bacterial cell membrane and cell wall fractions were isolated using a 10-mL culture of *L. casei* by following a previously described protocol [27]. The samples were resolved on a 20% polyacrylamide gel under denaturing conditions and then transferred to a 0.45-µm nitrocellulose membrane. After blocking overnight at 4°C with Blocking One (Nacalai, Kyoto, Japan), membranes were incubated at 4°C overnight with the mouse monoclonal antibody against FLAG (Sigma, MO, USA) at a dilution of 1:5000 in PBS. After washing the membranes with Tris-buffered saline with 0.05% Tween 20 (washing buffer), the target proteins were detected with alkaline phosphatase (AP)-labeled ReserveAP goat anti-mouse IgG (KPL, Gaithersburg, MD, USA) at a dilution of 1:1000 with 1% PBS containing 0.05% Tween 20 (PBST) as the diluent. Nitro-blue tetrazolium chloride and 5-bromo-4-chloro-3’-indolylphosphatase *p*-toluidine salt (Roche, Mannheim, Germany) were used as the AP colorimetric substrate for visualization.

### Animals

The animal experimental protocols were approved by the Institutional Animal Care and Use committee, and animal experiments were conducted according to the institutional ethical guidelines for animal experiments. Female C57BL/6 mice were obtained from Japan SLC, Inc. (Shizuoka, Japan). Mice were maintained in a specific pathogen-free manner and allowed to drink and eat *ad libitum*.

### Immunological Challenge using C. albicans

Before the survival studies for *C. albicans*-infected mice were undertaken, LD_50_ determination studies were conducted to determine the suitable lethal dose of *C. albicans* required for performing the survival tests. Mice were infected with *C. albicans* at concentrations ranging from 2.2 × 10^3^ cells/100 μL PBS to 2.2 × 10^6^ cells/100 μL PBS. Mouse survival was assessed over a period of 26 days after *C. albicans* administration.

To assess the vaccine efficacy, mice were infected with the determined LD_50_ dose of *C*. *albicans* resuspended in 100 μL PBS by tail-vein injection two weeks after the last immunization. Mice were observed daily for four weeks after the *C. albicans* challenge.

### Oral Administration Using Live L. casei Cells Displaying Eno1p on Their Surfaces

Seven-week-old female C57BL/6 mice (ten per group) were immunized with a solution of Eno1p-expressing *L. casei* cells or control cells (5.6 × 10^11^ cfu/400 µL). The immunization was administered to ten mice at weeks 0, 1, and 3 (priming) and then at week 7 (booster). The host strain *L. casei* 525 was used as a control. The *L. casei* cells were suspended in PBS (400 μL per animal) and administered via an intragastric tube after 2 h of fasting, once per day for 5 d per week. Blood samples were collected at week 9 from the tail vein to determine the titer of serum IgG.

### Endpoint Titer

The indirect enzyme-linked immunosorbent assay (ELISA) was performed for antibody analysis of antisera collected at week 9. Briefly, 96-well microtiter plates (Nalge Nunc International, Rochester, NY, USA) were coated with 50 μL/well of recombinant Eno1p (0.01 μg/μL). The plates were blocked with 1% BSA dissolved in PBS containing 0.05% Tween 20. Serially diluted antisera of mice, horseradish peroxidase (HRP)-labeled goat anti-mouse IgG (1:4000; Promega, Madison, WI, USA), and HRP substrate were sequentially added to the wells. After 20 min incubation at 25ºC, the reaction was stopped by adding 1 mol/L H_2_SO_4_, and the absorbance was measured at 450 nm (OD_450_) by using a microplate reader (Bio-Rad Laboratories Inc., Redmond, WA, USA). The serum IgG antibody titer was defined as the serum dilution that gave an OD_450_ value equal to 0.1.
